# MAGE-A1 in lung adenocarcinoma as a promising target of chimeric antigen receptor T cells

**DOI:** 10.1186/s13045-019-0793-7

**Published:** 2019-10-22

**Authors:** Yuan Mao, Weifei Fan, Hao Hu, Louqian Zhang, Jerod Michel, Yaqin Wu, Jun Wang, Lizhou Jia, Xiaojun Tang, Li Xu, Yan Chen, Jin Zhu, Zhenqing Feng, Lin Xu, Rong Yin, Qi Tang

**Affiliations:** 1 Department of Thoracic Surgery, Jiangsu Cancer Hospital, Jiangsu Institute of Cancer Research, Nanjing Medical University Affiliated Cancer Hospital, The Fourth Clinical College of Nanjing Medical University, Jiangsu Key Laboratory of Molecular and Translational Cancer Research, Nanjing, China; 2 NHC Key Laboratory of Antibody Technique, Jiangsu Key Laboratory of Cancer Biomarkers, Prevention and Treatment, Collaborative Innovation Center for Cancer Personalized Medicine, Nanjing Medical University, Nanjing, China; 3 Department of Hematology and Oncology, Department of Geriatric Lung Cancer Laboratory, Geriatric Hospital of Nanjing Medical University, Jiangsu Province Geriatric Hospital, Nanjing, China; 4 Department of Interventional Oncology, Renji Hospital, School of Medicine, Shanghai Jiao Tong University, Shanghai, China; 5 Department of Mathematics, Nanjing University of Aeronautics and Astronautics, Nanjing, China; 6 Department of Radiation Oncology, Jiangsu Cancer Hospital, Jiangsu Institute of Cancer Research, the Affiliated Cancer Hospital of Nanjing Medical University, Nanjing, China; 7 Department of Pathology, Jiangsu Cancer Hospital, Affiliated Cancer Hospital of Nanjing Medical University, Nanjing, China; 8 Huadong Medical Institute of Biotechniques, Nanjing, China; 9 Jiangsu Key Laboratory of Cancer Biomarkers, Prevention and Treatment, Collaborative Innovation Center for Cancer Personalized Medicine, Nanjing Medical University, Nanjing, China

**Keywords:** Lung adenocarcinoma, Cancer/testis antigen, MAGE, CAR-T cell

## Abstract

**Background:**

Cancer/testis antigens (CTAs) are a special type of tumor antigen and are believed to act as potential targets for cancer immunotherapy.

**Methods:**

In this study, we first screened a rational CTA MAGE-A1 for lung adenocarcinoma (LUAD) and explored the detailed characteristics of MAGE-A1 in LUAD development through a series of phenotypic experiments. Then, we developed a novel MAGE-A1-CAR-T cell (mCART) using lentiviral vector based on our previous MAGE-A1-scFv. The anti-tumor effects of this mCART were finally investigated in vitro and in vivo.

**Results:**

The results showed striking malignant behaviors of MAGE-A1 in LUAD development, which further validated the rationality of MAGE-A1 as an appropriate target for LUAD treatment. Then, the innovative mCART was successfully constructed, and mCART displayed encouraging tumor-inhibitory efficacy in LUAD cells and xenografts.

**Conclusions:**

Taken together, our data suggest that MAGE-A1 is a promising candidate marker for LUAD therapy and the MAGE-A1-specific CAR-T cell immunotherapy may be an effective strategy for the treatment of MAGE-A1-positive LUAD.

## Introduction

Lung cancer (LC) incidence has been continuously increasing for the past few years worldwide [1]. According to the latest data on cancer statistics, approximately 700,000 new cases of LC occurred in 2015, and LC has become the leading cause of cancer-related mortality in China [2]. Non-small cell lung cancer (NSCLC) accounts for 85% of all cases of LC, and lung adenocarcinoma (LUAD) is the most common histological type of NSCLC, accounting for nearly 40% of all LC-related deaths [3, 4]. Despite significant improvements in LUAD treatment, including surgery, chemotherapy, radiotherapy, and especially targeted therapy, the overall survival (OS) of LUAD is still frustrating. `The 5-year survival rate of patients with LUAD is less than 30% when it is treated in an early stage, and the OS rate decreases in patients with advanced LUAD because of its highly aggressive and metastatic characteristics [5, 6]. Therefore, it is of tremendous importance to develop novel therapeutic strategies for patients with LUAD.

Adoptive immunotherapy has been proven to have enormous potential in cancer treatment. In particular, chimeric antigen receptor-engineered T (CAR-T) cells have demonstrated antitumor activity, especially for hematological malignancies such as leukemia and lymphomas [7, 8]. For solid tumors, CAR-T therapy has also made progress, including in colorectal cancer [9], breast cancer [10], thyroid cancer [11], and head and neck cancer [12]. Although Feng et al. reported that targeting epidermal growth factor receptor (EGFR) in a clinical trial showed a good response in EGFR-expressing advanced relapsed/refractory NSCLC, and Li et al. described that CAR-glypican 3 T cells displayed promising therapeutic effectiveness for the treatment of patients with lung squamous cell carcinoma, research regarding LUAD is still limited. One of the most substantial impediments for the development of CAR-T therapy for solid tumors is the identification of tumor antigens. Currently, most tumor-associated antigens (TAAs) that are utilized as targets for CAR-T therapy are not tumor-specific, which means that they are expressed in both malignant and normal tissues [13]. A number of strategies have been developed to increase the controllability of CAR-T cells to minimize the on-target/off-tumor toxicities and typical side effects, such as cytokine release syndrome [14, 15]. Hence, for LUAD treatment, screening and identifying appropriate TAAs are essential steps.

Cancer/testis antigens (CTAs) constitute a type of special tumor antigen that is physiologically expressed in the germ cells of the testes as well as in a variety of malignant tumors but not in normal tissues [16]. Due to their unique immunogenic nature, CTAs are well known as ideal targets for cancer immunotherapy [17, 18]. However, the results of several clinical trials of therapeutic anticancer vaccines targeting CTAs were unsatisfactory [19–21]. Failures in clinical experiments clearly show the great importance of identifying appropriate and dependable CTAs to be used in the development of novel cancer immunotherapy strategies in the future, especially for CAR-T therapy because the tumor-limited expression of CTAs makes them the prime candidates for TAA selection. Previously, we described 876 CTA expression patterns in 19 cancer types by performing a comprehensive and multiplatform analysis through several publicly accessible databases [22].

In the present study, a rational CTA, MAGE-A1, for LUAD was screened after searching a database and conducting bioinformatics analyses. Then, phenotypic experiments were performed to verify the rationality of MAGE-A1 as an appropriate target for LUAD treatment. Moreover, a novel MAGE-A1-CAR-T cell (mCART) was constructed, and its anti-tumor effectiveness in vitro and in vivo was investigated.

## Materials and methods

### Database search and bioinformatics analyses

From the CTA database in our previous study [22], we retrieved LUAD-related data and screened candidate CTAs by score ranking (normalized expression > 3%). Then, we searched the GTEx Portal database (https://www.gtexportal.org) to further identify appropriate CTAs that are only expressed in testes and not in normal tissues. Next, we inspected the GeneCard database (http://www.genecards.org) to filter suitable CTAs that are expressed in the cytomembranes of cancer cells (expression confidence > 3). Moreover, we employed The Cancer Genome Atlas (TCGA) data (https://cancergenome.nih.gov) to validate the RNA expression levels of eligible CTAs in LUAD tissues and corresponding noncancerous tissues (expression fold change > 10). Finally, we checked the Human Protein Atlas database (http://www.proteinatlas.org) to ensure CTA protein expression in LC.

### Tissue sample collection

A tissue microarray (TMA) containing 90 cases of normal human tissue samples was purchased from Outdo Biotech Co., Ltd. (Shanghai, China). Simultaneously, five LUAD tissue samples and corresponding non-cancerous tissue samples were collected from the Department of Thoracic Surgery, Nanjing Medical University Affiliated Cancer Hospital. A TMA containing 93 cases of LUAD was also purchased from Outdo Biotech Co., Ltd. (Shanghai, China) [23]. Important clinical parameters were collected along with the LUAD TMA. Written informed consent was obtained from the patients for the publication of this study and the use of any accompanying images. The study protocol was approved by the Ethics Committee of Nanjing Medical University Affiliated Cancer Hospital, and all experiments were performed following the approved guidelines of Nanjing Medical University.

### Cell lines and reagents

Four LUAD cell lines (PC9, H1299, GLC82, A549) and the human embryonic kidney 293T cell line (HEK-293T) were preserved in our lab and enrolled in the present study. The human melanoma A375 cell line was purchased from the Cell Bank of the Chinese Academy of Sciences (Shanghai, China). The human normal bronchial epithelial (HBE) cell line was kindly provided by Professor. Erbao Zhang from the Department of Epidemiology and Biostatistics, Nanjing Medical University, to serve as the non-cancerous cell line. Peripheral blood mononuclear cells (PBMCs) derived from a healthy donor were collected by Ficoll-Hypaque density-gradient centrifugation conducted by the Jiangsu Blood Center. Medium with recombinant human interleukin-2 (IL-2) 300 U/ml was used for the expansion of T cells.

### One-step qPCR, western blotting, immunofluorescence, and immunohistochemistry analyses

MAGE-A1 expression was thoroughly examined in LUAD cell lines and tissue samples. For the qPCR, the sequences of the primers are listed in Additional file 7: Table S2. For the western blotting analysis, two types of primary monoclonal antibodies were obtained from Abcam (ab193330, ab243935, Abcam, Cambridge, MA, USA). The protocols of the qPCR test and western blotting analysis were described previously [24, 25]. The immunofluorescence test was conducted following the protocols described in our previous study [26]. Cells were incubated with FITC-labeled human anti-MAGE-A1 antibody (Abcam, ab212590) in the dark. 4′-6-diamidino-2-phenylindole (DAPI, Biotium, Hayward, CA) was used for nuclear staining. The ubiquitous Desmoglein 2 (DSG-2) was employed as a positive control (Abcam, ab150372). Immunohistochemistry (IHC) analysis was performed as previously described [27, 28]. TMA sections were incubated with mouse monoclonal anti-MAGE-A1 antibody (Abcam, ab193330). The secondary antibody used was horseradish peroxidase-conjugated anti-mouse antibody. Phosphate-buffered saline (PBS) was used as a negative control.

### Plasmid construction, lentivirus packaging, and infection

The overexpression and short-hairpin RNA (shRNA)-mediated knockdown lentivirus plasmids and packaging vectors were prepared as previously described [29]. Full-length MAGE-A1 was inserted into the lentivirus pLenti-EF1a-EGFP-P2A-Puro-CMV-MCS vector (Obio Technology, Co., Ltd., Shanghai, China). The detailed sequences of the three shRNAs and related siRNAs used in this study are listed in Additional file 7: Table S2. shRNA targeting MAGE-A1 (shMAGE) or scrambled shRNA (shCT) were cloned into pLKD-CMV-G&PR-U6-shRNA (Obio Technology). PC9 cells were then infected with MAGE-A1 overexpression (OEMAGE) or shMAGE viruses. After viral transfection, MAGE-A1 expression was evaluated by qPCR and western blotting analyses. Then, stable OEMAGE and shMAGE PC9 cell lines were confirmed by puromycin selection and prepared for further experiments.

### Cell proliferation, migration, and invasion assays

CCK-8, wound healing, and Transwell assays were performed in OEMAGE and shMAGE PC9 cell lines, respectively, to detect the malignant behaviors of MAGE-A1 in LUAD, including its effects on cell proliferation, cell migration, and cell invasion, as described before [30].

### Tumor growth assay in mice

Athymic 4-week-old BALB/c nude mice were purchased from SLAC Laboratory Animal Co., Ltd. (Shanghai, China) and kept under specific pathogen-free (SPF) conditions. In brief, 1.0 × 10^7^ PC9 (OEMAGE and shMAGE) cells were injected into nude mice subcutaneously. After inoculation, the tumor-bearing mice were observed, and tumor size was measured with a Vernier caliper. The subsequent procedures of the tumor growth assay in mice were described previously [26].

### mCAR construction

The MAGE-A1-CAR (mCAR) was designed to consist of a human CD8α leader, anti-MAGE-A1-scFv, CD8α hinge and transmembrane domain (CD8™), and CD137 and CD3ζ cytoplasmic domains [31, 32]. The anti-MAGE-A1 scFv was determined in our previous study [33], and the detailed amino acid sequence is shown in Additional file 8: Table S3. The fragments encoding the CD8α leader, anti-MAGE-A1 scFv, CD8™, and CD137-CD3ζ were produced by PCR and cloned into the EcoRI and XbaI sites of the lentiviral expression vector pLVX-IRES-ZsGreen (Clontech, USA). All positive clones were confirmed by sequencing analysis.

### Lentivirus production

For lentivirus production, HEK-293T cells were co-transfected with mCAR vector, pMD2.G plasmid (Invitrogen, Carlsbad, CA, USA) and packaging psPAX2 plasmid (Invitrogen). Supernatants containing the lentivirus were collected 48 h and 72 h later. After filtration through a 0.45-μm filter, the lentivirus supernatant was concentrated 30-fold by ultracentrifugation (Amicon Ultra 100 kD, Millipore, USA). 293T cells transfected with CD19-CAR (unrelated-CAR) and untransfected 293T cells (blank) were employed as controls. Then, CD3ζ was selected as the target to test mCAR expression after 293T cell transfection by western blotting analysis.

### Sandwich ELISA assay

A sandwich ELISA was performed to evaluate the binding ability of mCAR to MAGE-A1 as described before [34]. Briefly, 96-well plates were seeded with transfected 293T cells (mCAR and unrelated-CAR). Untransfected 293T cells (blank) were used as a negative control. Then, each well was washed and MAGE-A1 antigens were added (Novus Biologicals, Littleton, CO, USA) at different dilutions. Then, the supernatants were collected and added to another 96-well plate, which was preliminarily coated with anti-MAGE-A1 rabbit polyclonal antibody (LS-C327797-200, LifeSpan BioSciences, Seattle, WA, USA), followed by the addition of a primary anti-MAGE-A1 mouse monoclonal antibody (LS-C25368-100, LifeSpan BioSciences) and a secondary anti-mouse antibody. After washing, the optical density at 450 nm (OD450) was measured with an automatic microplate reader (Thermo Fisher Scientific, USA). The supernatant lentivirus titers were detected following the protocol described previously [35, 36].

### T cell collection and mCART preparation

PBMCs were separated from 10 mL of peripheral blood from a healthy volunteer using lymphocyte separation medium. PBMCs were activated in 24-well plates coated with anti-human CD3 (Life Technologies, Mountain View, CA, USA) and anti-human CD28 antibodies (Life Technologies) at day 0. After 48 h, IL-2 (300 U/mL) was added to stimulate the expansion of the T cells. After 72 h, T cells were transfected with the mCAR lentivirus. Unrelated-CART and control T cells (T) served as controls. At day 7, all T cells were harvested, and the details of the mCART activity and characteristics were examined by flow cytometry. Briefly, the transfection efficiency of T cells expressing CAR was tested by direct GFP (ZsGreen) fluorescence and MAGE-A1-PE staining. Phenotypic characterization and activation of the T cells were determined by staining with CD3, CD4, and CD8. Flow cytometry was performed on a BD FACSCelesta flow cytometer. Data were graphed using FlowJo 7.6 software (Ashland, OR, USA).

### Detection of the anti-tumor effectiveness of mCART in vitro

Antitumor activity was quantified by LDH release assay, as described previously [37]. mCART, unrelated-CART, and T were co-cultured with LUAD cell lines (H1299, PC9, PC9(sh)) at different ratios (20:1, 10:1, 5:1, 2:1). Then, mCART was co-cultured with different LUAD cell lines (PC9, H1299, GLC82, A549) at a fixed ratio (10:1). The HBE cell line was employed as a control. Unrelated-CART representsCD19-CAR-T cells that are produced and preserved in our lab. The supernatant was analyzed for IFN-γ and IL-2 production using the related ELISA assay kits (eBioscience, San Diego, CA, USA) following the manufacturer’s protocols.

### Detection of the anti-tumor effectiveness of mCART in vivo

Athymic BALB/c nude mice were purchased from SLAC. For LUAD xenograft model establishment and bioluminescent imaging of in vivo tumors, mice were injected with luciferase-expressing H1299 cells with matrix. After inoculation, mice were divided randomly into three groups (mCART group, unrelated-CART group, T group). Treatment was initiated when the xenografts reached volumes of approximately 100 mm^3^, and mice underwent fully myeloablative radiation. On days 0, 3, and 6, mice received intravenous treatment with mCART (1 × 10^7^), unrelated-CART and T cells. The tumor diameter was measured, and the tumor volume was calculated as described previously [26]. For bioluminescent imaging, mice were injected intraperitoneally with D-luciferin (Gold Biotechnology, St. Louis, MO, USA), and images were recorded on days 2, 5, 8, 13, and 20 by utilizing an IVIS Lumina II (PerkinElmer, Hopkinton, MA, USA). On day 27, all mice were killed, and the xenograft tumors were removed for further analysis. Specifically, CD3 expression was detected by IHC analysis using a primary rabbit monoclonal antibody (Abcam, ab16669). The detailed protocol of IHC analysis was described previously.

## Results

### MAGE-A1 is determined to be a suitable candidate CTA for LUAD

**Fig. 1:**
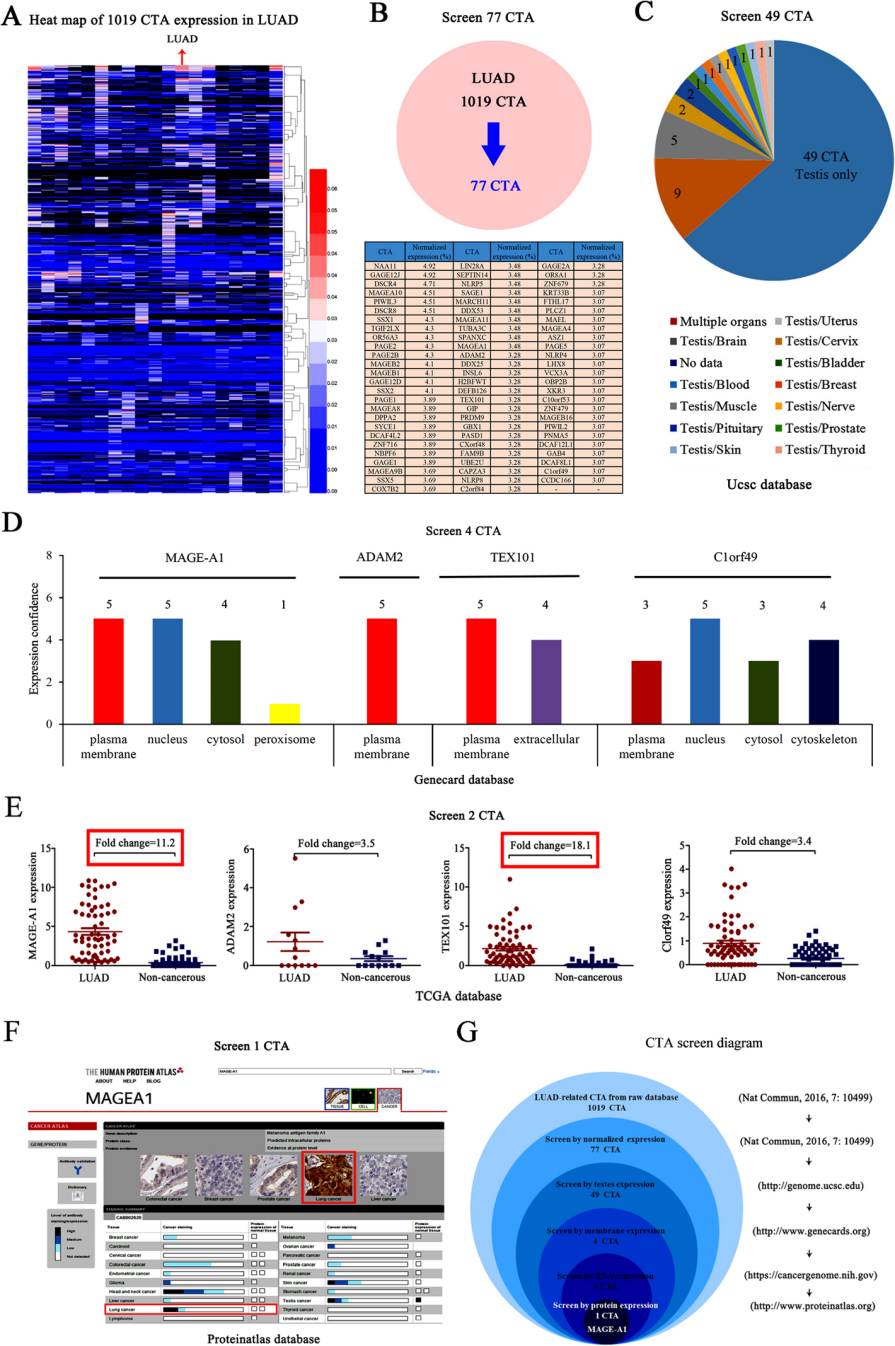
Bioinformatics analyses for the CTA screening. **a** Raw data were retrieved, and a heat map of the expression of 1019 CTAs in LUAD was created. **b** A total of 77 candidate CTAs were screened based on score ranking (normalized expression fold > 3%). **c** In total, 49 candidate CTAs that were exclusively expressed in the testis were screened (GTEx Portal database). **d** Four CTAs (MAGE-A1, ADAM2, TEX101, and Clorf49) that were expressed in the cytomembranes of cancer cells (expression confidence > 3) were screened (GeneCard database). **e** Two CTAs (MAGE-A1 and TEX101) had elevated RNA expression in LUAD tissues compared with the corresponding noncancerous tissues (expression fold change > 10, marked by a red box) and were selected (TCGA database). **f** One CTA (MAGE-A1) that was positively expressed in LC was screened and is marked by a red box (Human Protein Atlas database). **g** The screening diagram summarizes the entire process by which MAGE-A1 was finally identified as an appropriate CTA from among the original 1019 CTAs

First, we retrieved raw LUAD-related data and created a CTA expression heat map with a total of 1019 CTAs (Fig. 1a, Additional file 6: Table S1). Then, we screened 77 candidate CTAs by score ranking (normalized expression > 3%) (Fig. 1b, Additional file 6: Table S1). Subsequently, we searched the GTEx Portal database to further identify 49 CTAs that were only expressed in the testes and not in normal tissues (Fig. 1c, Additional file 1: Figure S1, Additional file 6: Table S1). After that, we inspected the GeneCard database to identify four suitable CTAs that were expressed in the cytomembranes of cancer cells (expression confidence > 3) because the cytomembrane expression of CTAs is important for the construction of CAR-T cells (Fig. 1d, Additional file 2: Figure S2, Additional file 6: Table S1). Moreover, we employed TCGA data to validate 2 CTAs, of which the RNA expression in LUAD tissues was markedly higher than that in the corresponding non-cancerous tissues (expression fold change > 10) (Fig. 1e, Additional file 6: Table S1). In addition, we consulted the Human Protein Atlas database to ensure that the qualified CTAs are positively expressed in LC (Fig. 1f, Additional file 6: Table S1). Finally, MAGE-A1 was selected as the appropriate LUAD-associated CTA from among the original 1019 CTAs (Fig. 1g).

### MAGE-A1 is highly expressed in LUAD cell lines

**Fig. 2:**
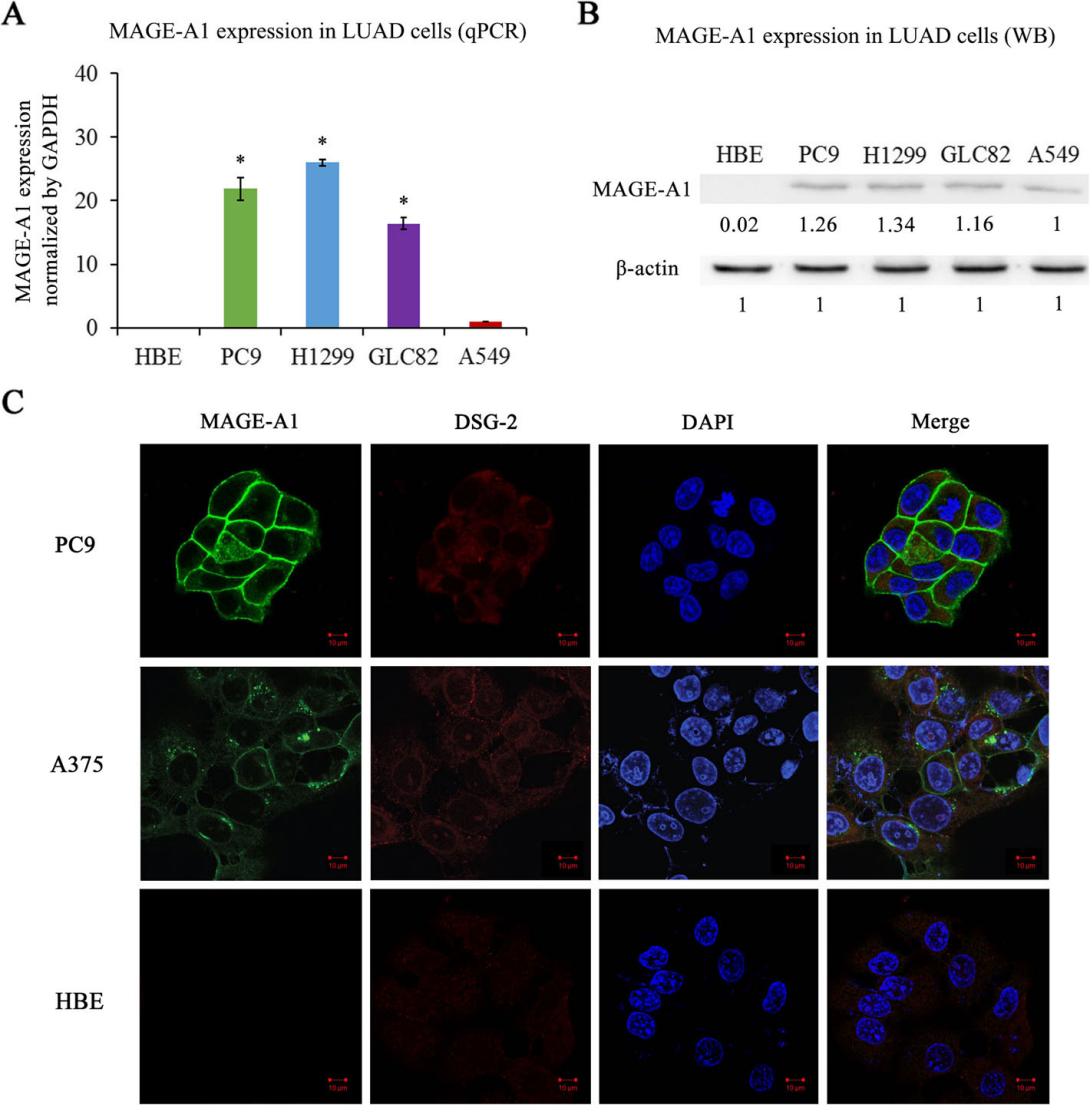
The detection of MAGE-A1 expression in LUAD cell lines. **a**, **b** Detection of MAGE-A1 expression in LUAD cell lines (PC9, H1299, GLC82, A549) by qPCR and western blotting analyses. The human normal bronchial epithelial (HBE) cell line was used as a non-cancerous control cell line. *Significant difference of MAGE-A1 expression in LUAD cell lines compared with HBE cells. *p* < 0.05. **c** Immunofluorescence assay revealed that MAGE-A1 could be stained in MAGE-A1-positive PC9 cell but not in MAGE-A1-negative HBE cell. The human melanoma A375 cell line was employed as a positive control and MAGE-A1-positive staining could also be observed in A375 cell line. Strong staining of MAGE-A1 was mainly localized in the cytomembrane while relatively weak staining of MAGE-A1 was observed in cytoplasm of cancer cells. Green, MAGE-A1 staining; red, DSG-2 staining; blue, nuclear staining

To confirm the expression of MAGE-A1 in LUAD, qPCR and western blotting analyses were performed in LUAD cell lines. In four LUAD cell lines, the results of both qPCR and western blotting analyses showed that MAGE-A1 expression was significantly higher than that in the normal HBE cell line (Fig. 2a, b). Immunofluorescence assay revealed that MAGE-A1 could be stained in MAGE-A1-positive PC9 cell but not in MAGE-A1-negative HBE cell. The human melanoma A375 cell line was employed as a positive control and MAGE-A1-positive staining could also be observed in A375 cell line. Strong staining of MAGE-A1 was mainly localized in the cytomembrane while relatively weak staining of MAGE-A1 was observed in the cytoplasm of cancer cells (Fig. 2c).

### MAGE-A1 is dominantly expressed in LUAD tissues

**Fig. 3:**
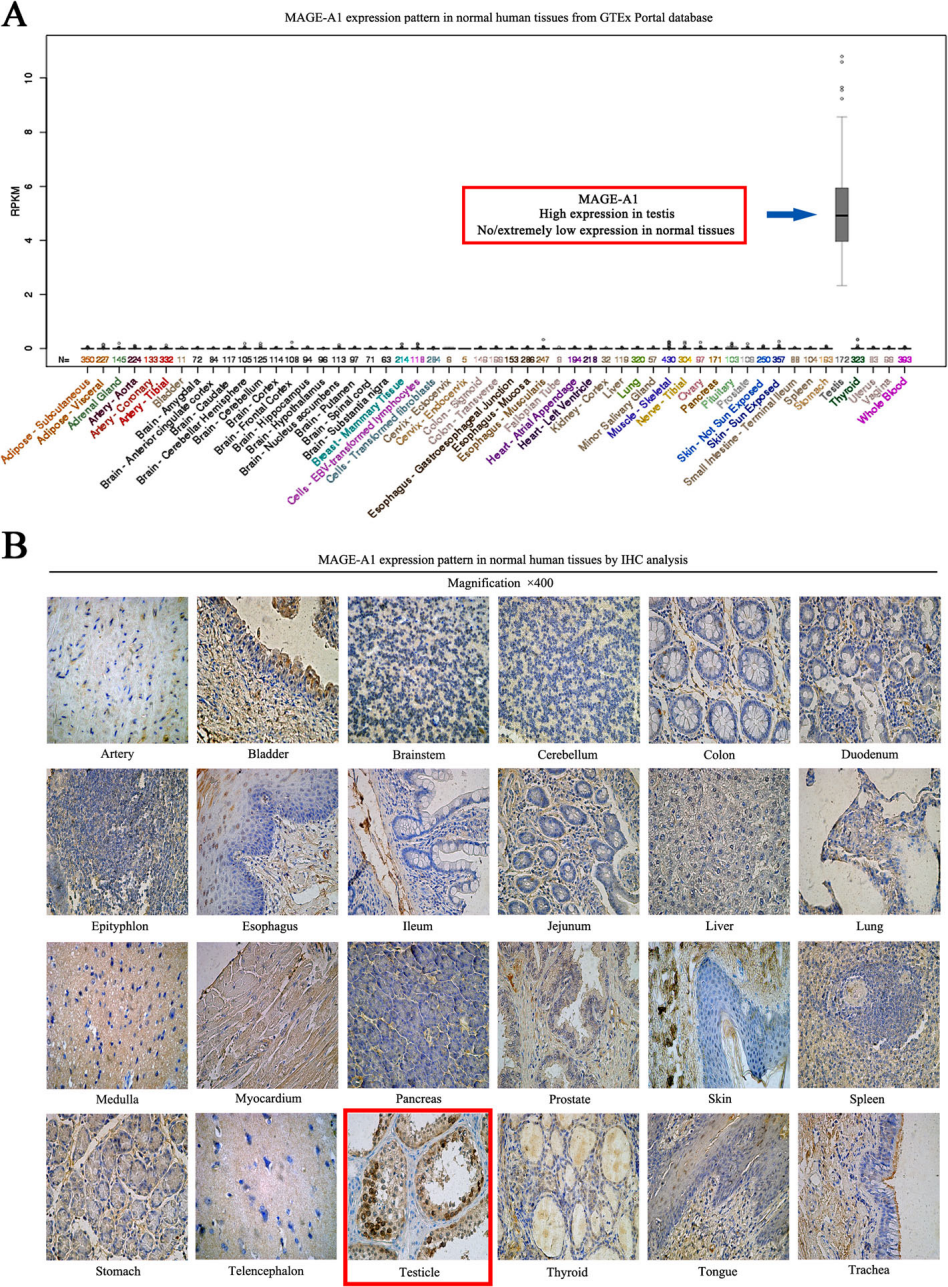
The detection of MAGE-A1 expression in normal human tissues. **a** GTEx Portal database illustrated the expression mode of MAGE-A1 (red box) in normal human tissue, and the data showed that MAGE-A1 was mostly expressed in human testis. **b** IHC analysis in normal human TMA demonstrated that the MAGE-A1 expression was largely witnessed in human testicle samples (red box) while rarely observed in other human tissue samples, including the artery, bladder, brainstem, cerebellum, colon, duodenum, epityphlon, esophagus, ileum, jejunum, liver, lung, medulla, myocardium, pancreas, prostate, skin, spleen, stomach, telencephalon, thyroid, tongue, and trachea

**Fig. 4:**
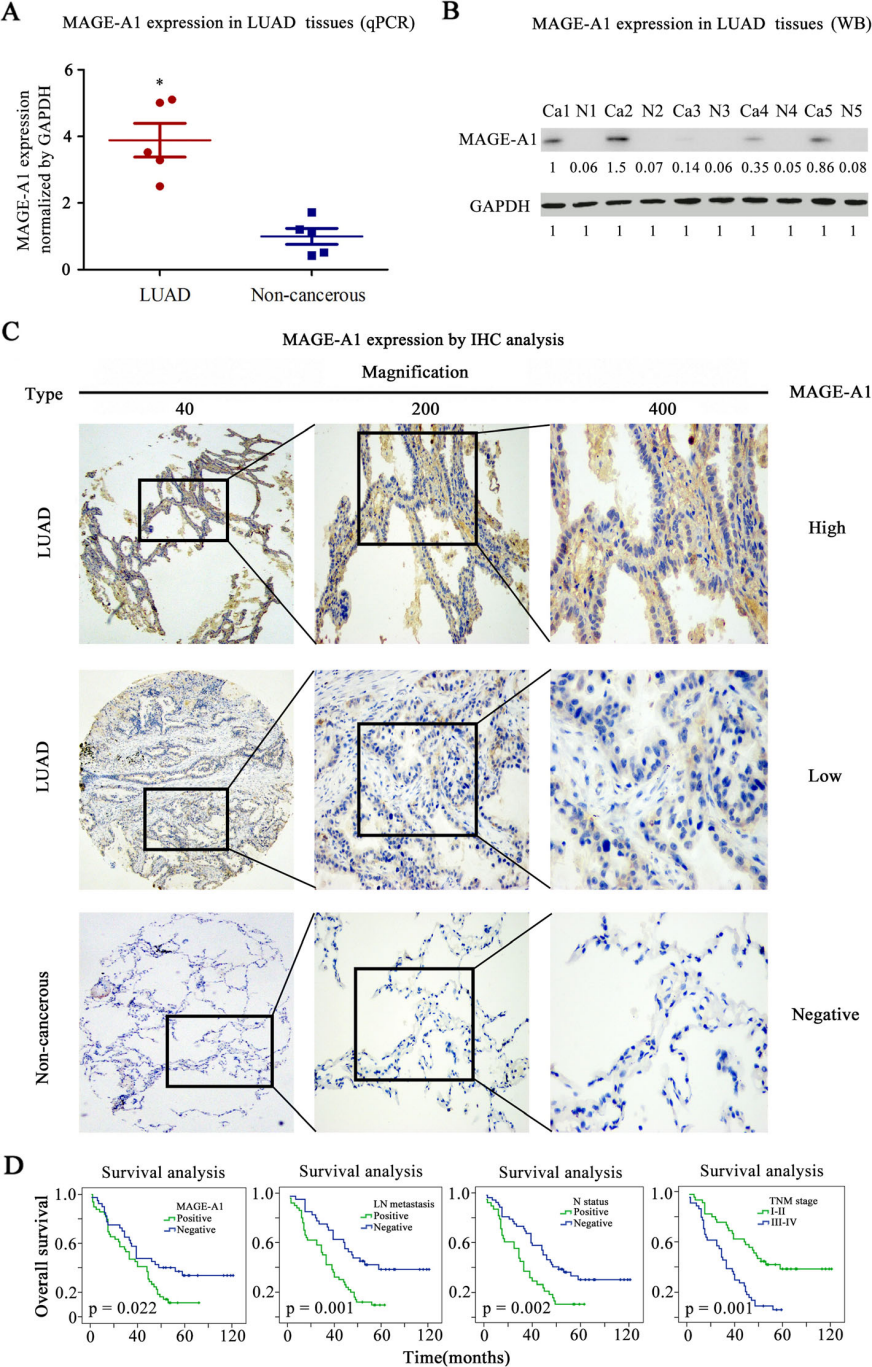
The detection of MAGE-A1 expression in LUAD tissues. **a**, **b** qPCR and WB tests in five LUAD and non-cancerous tissue samples showed that the expression of MAGE-A1 in LUAD was elevated compared with that in non-cancerous tissues. *Significant difference of MAGE-A1 expression in LUAD tissue samples compared with non-cancerous tissue samples. *p* < 0.05. **c** Detection of MAGE-A1 expression in a tissue microarray (TMA) containing 92 LUAD samples by immunohistochemistry (IHC) analysis. Positive staining of MAGE-A1 was mainly located in the cytoplasm of LUAD cells. **d** The survival analysis and Kaplan-Meier curve illustrated that positive MAGE-A1 expression (*p* = 0.022), positive lymph node metastasis (*p* = 0.001), positive N status (*p* = 0.002), and advanced TNM stage (*p* = 0.001) were significantly correlated with a poor prognosis of patients with LUAD

We searched GTEx Portal database to preliminarily detect the expression mode of MAGE-A1 in normal human tissue and the data showed that MAGE-A1 was mostly expressed in human testis (Fig. 3a). Further, IHC analysis in normal human TMA confirmed that the MAGE-A1 expression was largely witnessed in human testicle samples while rarely observed in other human tissue samples (Fig. 3b). Then, we collected five LUAD and noncancerous tissue samples, and the data from qPCR and WB tests showed that the expression of MAGE-A1 in LUAD was elevated compared with that in non-cancerous tissues (Fig. 4a, b). After IHC analysis in LUAD TMA, 4 samples of LUAD and 9 samples of non-cancerous tissue in TMA were missing. The results of IHC analysis demonstrated that high MAGE-A1 expression was detected in 49 of 89 (44%) LUAD tissues compared with14 of 78 (18%) non-cancerous tissues, and the difference was highly significant (*χ*^*2*^ = 24.36, *p* = 0.001). The IHC staining for MAGE-A1 expression and its relationships with important clinical characteristics in LUAD patients are presented in Fig. 4c and Table 1. A high level of MAGE-A1 expression was significantly correlated with tumor diameter (*p* = 0.023) and N status (*p* = 0.031). A survival analysis was performed, and the results illustrated that MAGE-A1 expression was critically associated with OS in patients with LUAD (*p* = 0.022) but was not an independent prognostic predictor (*p* = 0.087) (Fig. 4d and Table 2).

**Table 1:** Correlation of high MAGE-A1 protein expression with clinicopathological characteristics in 89 LUAD

Groups	No.	MAGE-A1		*χ* ^*2*^	*P* value
		+	%		
Gender					
Male	49	28		0.1918	0.661
Female	40	21			
Age					
≥ 60 years	56	31		0.0055	0.941
< 60 years	33	18			
Tumor diameter					
≥ 3 cm	56	36		5.1993	0.023*
< 3 cm	33	13			
Pathological grade					
Grade I–II	64	36		0.1312	0.717
Grade III	25	13			
Lymph node metastasis					
Positive	48	30		2.3246	0.127
Negative	39	18			
Insufficient data	2	1			
T status					
T1–T2	68	35		1.4974	0.221
T3–T4	21	14			
N status					
Positive	36	25		4.6647	0.031*
Negative	50	23			
Insufficient data	3	1			
M status					
Positive	1	1		0.8051	0.370
Negative	87	48			
Insufficient data	1	0			
TNM stage					
Stage I–II	44	21		2.3890	0.122
Stage III–IV	42	27			
Insufficient data	3	1			

*p < 0.05

**Table 2:** Univariate and multivariate analysis of prognostic factors for overall survival in LUAD patients

	Univariate analysis			Multivariate analysis		
	HR	*p* value	95% CI	HR	*p* value	95% CI
MAGE-A1 expression						
High versus low	1.78	0.022*	1.09–2.93	1.58	0.087	0.93–2.68
Gender						
Male versus female	1.38	0.183	0.86–2.20			
Age						
≥ 60 years versus < 60 years	0.96	0.861	0.59–1.55			
Tumor diameter						
≥ 3 cm versus < 3 cm	1.54	0.090	0.93–2.55			
Pathological grade						
Grade I–II versus grade III	0.88	0.633	0.53–1.47			
Lymph node metastasis						
Positive versus negative	2.47	0.001*	1.49–4.09	1.02	0.950	0.44–2.42
T status						
T1–T2 versus T3–T4	0.71	0.196	0.42–1.20			
N status						
Positive versus negative	2.11	0.002*	1.31–3.41	1.42	0.265	0.77–2.65
M status						
Positive versus negative	1.09	0.930	0.15–7.90			
TNM stage						
Stage I–II versus stage III–IV	0.36	0.001*	0.21–0.59	0.45	0.029*	0.22–0.92

*HR* hazard ration, *CI* confidence interval, *LUAD* lung adenocarcinoma

*p < 0.05

### MAGE-A1 is positively associated with malignant behaviors of LUAD

**Fig. 5:**
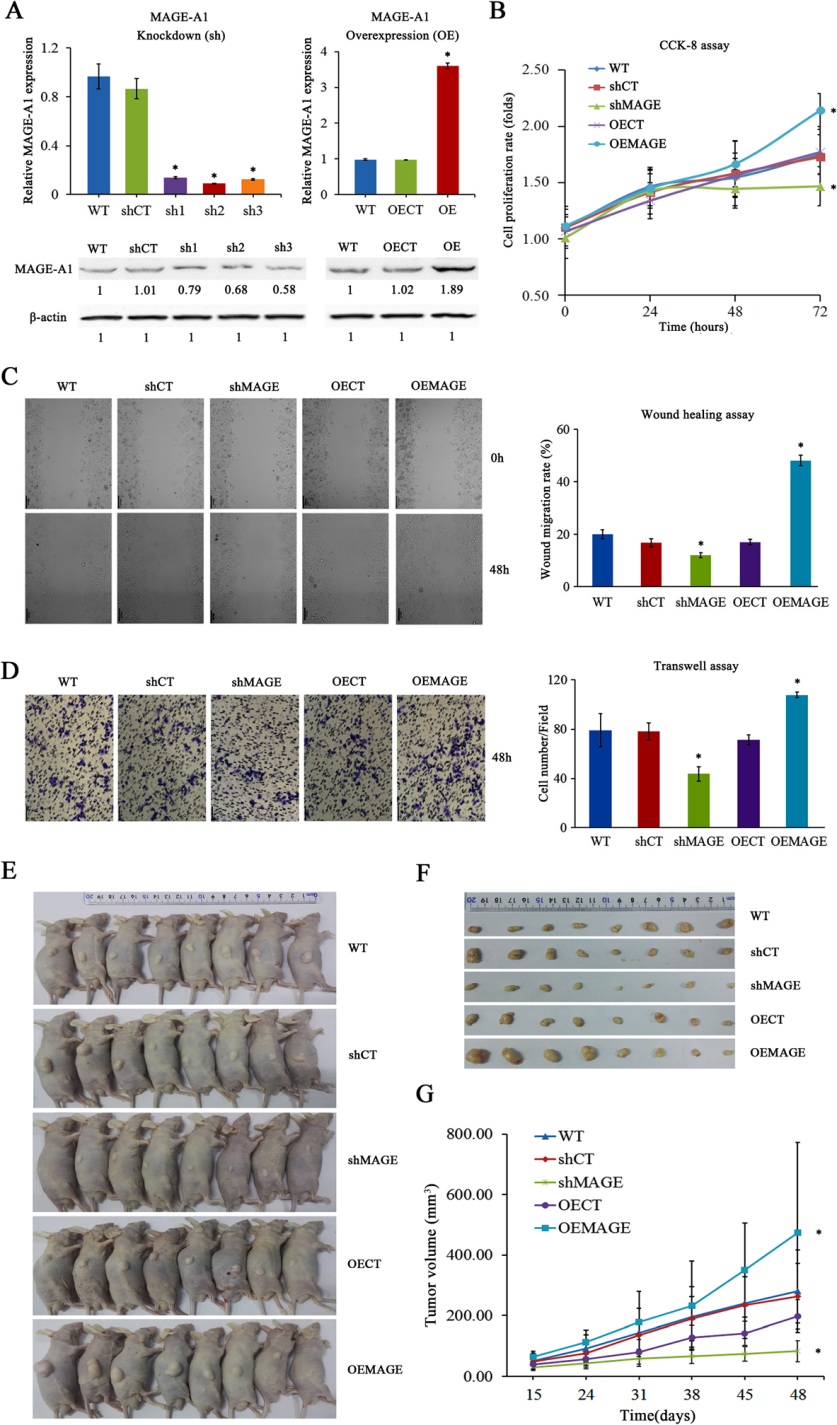
The investigation of MAGE-A1 activity in the development of LUAD in vitro and in vivo. **a** MAGE-A1 knockdown (shMAGE) and MAGE-A1 overexpression (OEMAGE) models were successfully constructed using the PC9 cell line. For shMAGE, qPCR and western blotting analyses showed that MAGE-A1 expression levels in shMAGE1 (sh1), shMAGE2 (sh2), and shMAGE3 (sh3) were significantly reduced. *Significant difference in MAGE-A1 expression in shMAGE cell line compared with the wild-type (WT) cell line. *p* < 0.05. For OEMAGE, qPCR and western blotting analyses showed that the MAGE-A1 expression level in OEMAGE (OE) was significantly elevated. *Significant difference in MAGE-A1 expression in the OEMAGE cell line compared with the WT cell line. *p* < 0.05. **b**–**d** CCK-8, wound healing, and transwell assays demonstrated that shMAGE drastically inhibited PC9 cell proliferation, migration, and invasion, while OEMAGE significantly augmented PC9 cell proliferation, migration, and invasion in vitro. *Significant difference in cell proliferation, migration, and invasion in shMAGE or OEMAGE cell lines compared with WT cell lines. **e**, **f** Xenograft tumors developed from OEMAGE cells grew significantly faster than those developed from shMAGE cells. **g** The volume of shMAGE tumors was much smaller than that of OEMAGE tumors at 48 days after cell inoculation. *Significant difference in tumor volume in tumors from shMAGE or OEMAGE cell lines compared with tumors from WT cell lines

Because MAGE-A1 was upregulated in LUAD, the biological role of MAGE-A1was explored by CCK-8, wound healing and transwell assays in the PC9 cell line. As shown in Fig. 5a, we successfully constructed MAGE-A1 knockdown (shMAGE) and MAGE-A1 overexpression (OEMAGE) models. shMAGE drastically inhibited PC9 cell proliferation, migration, and invasion, while OEMAGE significantly augmented PC9 cell proliferation, migration, and invasion (Fig. 5b–d). Then, shMAGE and OEMAGE PC9 cells were subcutaneously injected into nude mice. As shown in Fig. 5e, the xenograft tumors that developed from OEMAGE PC9 cells grew significantly faster than those that developed from shMAGE PC9 cells. Consistently, the weight (Fig. 5f, Additional file 3: Figure S3) and volume (Fig. 5g) of MAGE-A1 knockdown tumors were much lighter and smaller than those of MAGE-A1 overexpression tumors at 48 days after cell inoculation. These results indicate the promotional function of MAGE-A1 in LUAD tumorigenesis.

### Generation and characterization of mCART

**Fig. 6:**
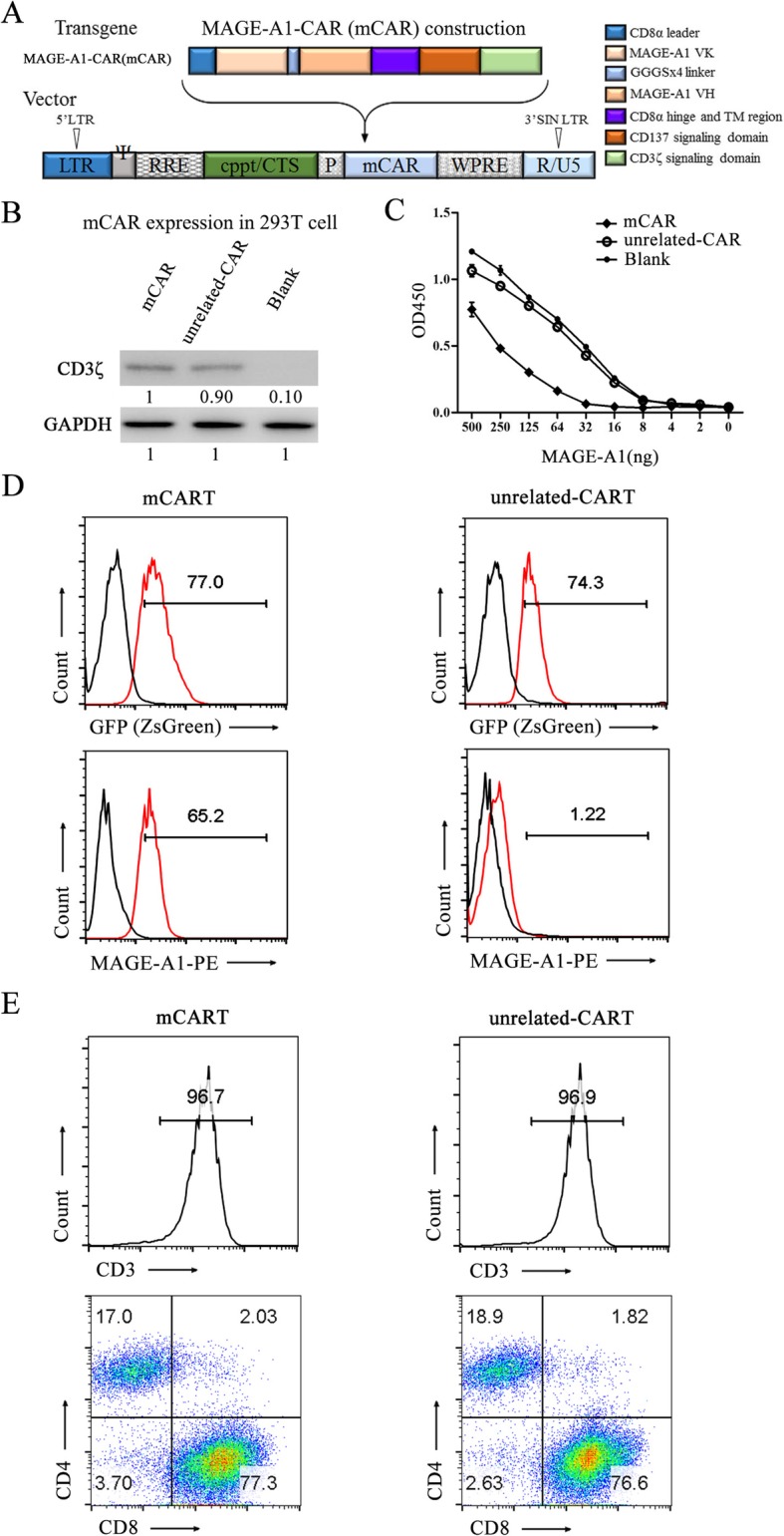
MAGE-A1-CAR-T cell construction and identification. **a** The lentiviral vector construct of MAGE-A1-CAR (mCAR), the TM transmembrane portion. The mCAR is composed of the MAGE-A1-scFv linked to a human CD8a leader, CD8a hinge, and the transmembrane domain fused to an intracellular signaling domain derived from human CD137 and CD3ζ. **b** CD3ζ was detected by western blotting in HEK-293T cells transfected with mCAR. HEK-293T cells transfected with an unrelated-CAR were used as a positive control. Untransfected 293T cells (blank) were employed as a negative control. **c** A sandwich ELISA was performed to evaluate the binding ability of mCAR to MAGE-A1. 293T cells transfected with mCAR and unrelated-CAR were enrolled. Untransfected 293T cells were employed as control (blank). **d** The transfection efficiencies of mCART and unrelated-CART by GFP (ZsGreen) were 77.0% and 74.3%, respectively. In comparison, the transfection efficiencies of mCART and unrelated-CART by MAGE-A1-PE staining were 65.2% and 1.22%, respectively. **e** Flow cytometry analysis showed that CD3-positive, CD4-positive, and CD8-positive T cells in mCART were obtained from PBMCs by magnetic bead separation, activated by CD3/CD28 co-stimulation and transfected by mCAR lentivirus. An unrelated-CART was used as a positive control

The structure of mCAR is shown in Fig. 6a, consisting of a signal peptide leader sequence of CD8α, MAGE-A1-scFv, the hinge spacer, and the transmembrane region of CD8α, the costimulatory molecule CD137 intracellular domain and the CD3ζ signaling moieties. Then, the western blotting analysis was used to detect CD3ζ expression to illustrate the outcome of mCAR generation, and the results confirmed the successful construction and expression of mCAR in 293T cells after transfection. 293T cells transfected with unrelated-CAR were used as a positive control, and 293T cells without transfection were employed as a negative control (Fig. 6b). The results of the sandwich ELISA further implied that compared with 293T cells transfected with unrelated-CAR or untransfected 293T cells, 293T cells transfected with mCAR could specifically bind the uncombined MAGE-A1 antigen, which indicates that the MAGE-A1-scFv contained in mCAR could expectedly recognize MAGE-A1 antigen (Fig. 6c). The lentivirus titer was 1 × 10^8^ TU/mL after detection (Additional file 4: Figure S4). Then, the lentiviral vector encoding mCAR or unrelated-CAR was used to transfect CD3/CD28-activated T cells from a healthy donor. After 7 days of stimulation, fluorescence-activated cell sorting (FACS) analysis demonstrated that the transfection efficiencies of mCART and unrelated-CART by GFP (ZsGreen) were 77.0% and 74.3%, respectively. In comparison, MAGE-A1-PE staining showed that the transfection efficiency of mCART was 65.2%, which was significantly higher than that of unrelated-CART (1.22%) (Fig. 6d). Then, the phenotype of the stimulated T cells after transfection was further determined by FCM analysis. One week after co-culture in the presence of CD3/CD28 antibodies, more than 90% of the sorted cells were CD3-positive, and 80% of the sorted cells were CD8-positive in mCART as well as in unrelated-CART (Fig. 6e). The results strongly suggested that T cells were successfully infected with the lentiviral vector containing the mCAR and that the characteristic mCART was verified.

### mCART exerts anti-tumor activity against LUAD cells in vitro

**Fig. 7:**
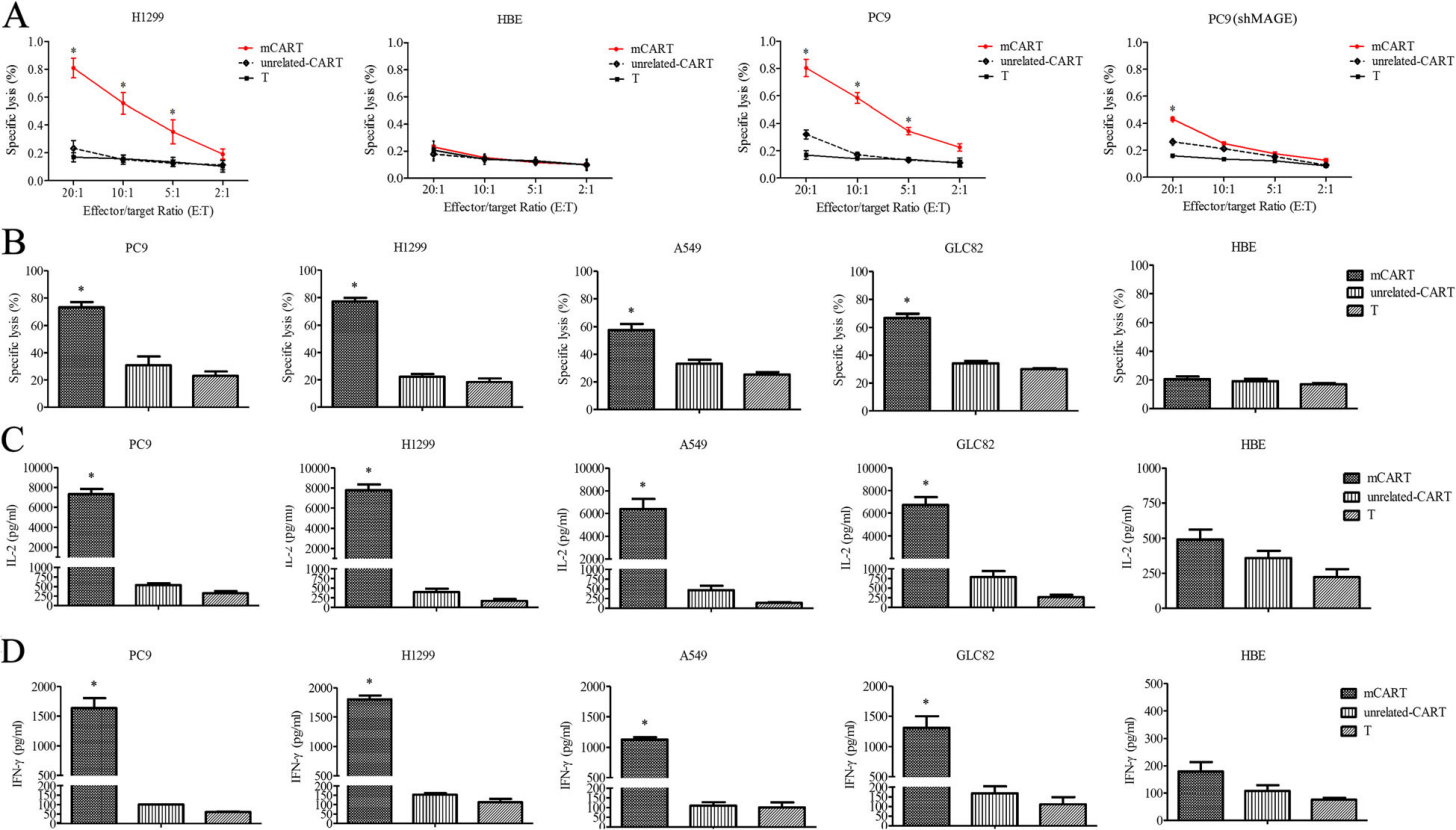
Anti-tumor activity of mCART against LUAD was explored by LDH release assay in vitro. **a** The tumor-inhibitory rate of mCART in the H1299 and PC9 (both MAGE-A1 positive) cell lines was progressively upregulated along with the increase in the E:T ratio of mCART. The 20:1 ratio of mCART showed the most effective cell killing activity. In comparison, mCART was barely able to kill MAGE-A1-negative cell lines (HBE and PC (shMAGE)). *Significant difference in tumor-inhibitory rate in the mCART group compared with the T group. **b** A fixed 10:1 E:T ratio of mCART also demonstrated significant tumor-inhibitory efficacy in all MAGE-A1-positive LUAD cell lines. For all the cell viability assays, unrelated-CART and T showed no cell-killing activities, regardless of the E:T ratio selected or the cell type used.*Significant difference in tumor-inhibitory rate in the mCART group compared with the T group. **c** and **d** IFN-γ and IL-2 expression were detected when mCART was co-incubated with LUAD cells. The 10:1 E:T ratio of mCART was co-cultured with four different cell lines. After culturing, a larger amount of IFN-γ and IL-2 was released by mCART, and their release was highly associated with the level of MAGE-A1 expression in the LUAD cells. In contrast, the release of IFN-γ and IL-2 remained unchanged in unrelated-CART and T cells. *Significant difference in IFN-γ and IL-2 expression in the mCART group compared with the T group

When co-cultured with LUAD cell lines, mCART mediated significant cell-killing activity in a dose-dependent manner. As shown in Fig. 7a, the tumor-inhibitory rate of mCART in the H1299 and PC9 (MAGE-A1 positive) cell lines was progressively upregulated along with the increase in the E:T ratio of mCART. mCART, with a 20:1 ratio, showed the most effective cell killing activity. In comparison, mCART showed highly ineffective cell-killing ability in MAGE-A1-negative cell lines (HBE and PC (shMAGE)), even though the E:T ratio of mCART was elevated. Then, a fixed E:T ratio of mCART was chosen, and mCART also illustrated significant tumor-inhibitory efficacy for all MAGE-A1-positive LUAD cell lines (Fig. 7b). In all the cell viability assays, unrelated-CART and T showed no cell-killing activities, regardless of the E:T ratio selected or the cell type used. Moreover, mCART co-incubated with LUAD cells caused a large release of cytokines, including IFN-γ and IL-2. In contrast, the release of IFN-γ and IL2 remained unchanged in the unrelated-CART group and T group (Fig. 7c, d). The above data clearly showed the potent tumor-inhibitory role of mCART in MAGE-A1-positive LUAD cells.

### mCART exerts anti-tumor activity against LUAD xenografts in vivo

**Fig. 8:**
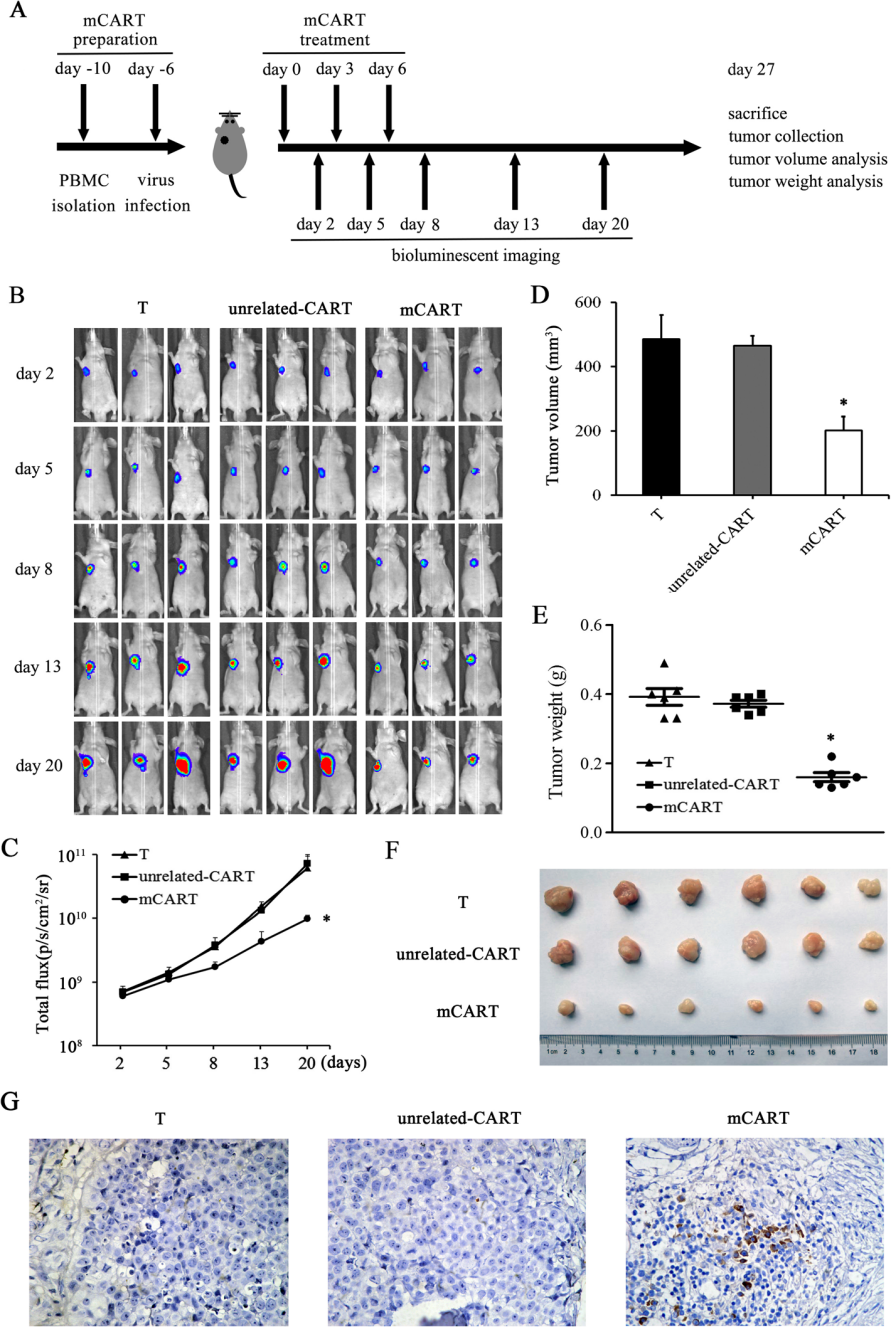
The anti-tumor activity of mCART against LUAD was investigated by mouse xenografts in vivo*.* **a** Flow diagram of the in vivo test. For mCART preparation, PBMCs from a healthy donor were collected on day 10, and lentivirus infection was performed on day 6. Mice were subcutaneously implanted with luciferase-expressing H1299 cells until the tumor volume reached approximately 100 mm^3^ and then randomly divided into three groups (mCART, unrelated-CART, and T). On days 0, 3, and 6, mice received intravenous treatment with mCART (1 × 10^7^), unrelated-CART, and T therapy. On days 2, 5, 8, 13, and 20, bioluminescent images were recorded. On day 27, all mice were killed, and the tumors from each animal were removed, measured, and weighed individually. **b**, **c** Serial bioluminescence imaging and tumor signal in mice was recorded to follow tumor progression. *Significant difference in bioluminescence imaging in the mCART group compared with that in the T group. **d** Serial volume of xenograft tumors. *Significant difference in the volume of xenograft tumors in the mCART group compared with the T group. **e**, **f** Comparison of xenograft tumor weight and morphology on day 27. *Significant difference in weight of xenograft tumors in the mCART group compared with the T group. **g** Comparison of CD3 expression in xenograft tumors by IHC analysis

The xenograft tumor models produced by inoculation of athymic nude mice with H1299 cells were constructed to investigate the anti-tumor function of mCART, following the protocol shown in Fig. 8a. Bioluminescent imaging of xenograft LUAD derived from luciferase-expressing H1299 cells illustrated a substantial effect on the tumors upon mCART administration (Fig. 8b, c). The tumor growth curve also confirmed that mCART led to a progressive and critical reduction in tumor burden (Additional file 5: Figure S5A). The mean body weight of nude mice in the three groups showed no significant difference (Additional file 5: Figure S5B). On day 27, all mice were sacrificed, and the xenograft tumors were removed and analyzed. The tumor volume (Fig. 8d), weight (Fig. 8e), and morphology (Fig. 8f) further confirmed that mCART can specifically target and significantly inhibit MAGE-A1-positive LUAD xenograft growth in vivo. IHC analysis of CD3 expression in xenograft tumors highly proved that mCART was able to infiltrate into tumors and exert tumor-inhibitory effectiveness (Fig. 8g).

## Discussion

Notwithstanding the noteworthy success of CAR-T cells for the treatment of hematologic malignancies, the efficacy of CAR-T cells in the treatment of solid tumors is less effective due to obstacles and limitations, such as off-target and off-tumor toxicity, incompetence of infiltration and persistence, and immunosuppression in the tumor microenvironment [38]. Further development of CAR-T therapy in solid tumors needs to overcome many impediments. First and foremost, identifying a suitable target antigen is one of the greatest challenges in the development of CAR-T therapy for solid tumors [39]. Given the exceptional properties of CTAs, it is logical to look for an appropriate antigen from among the CTAs for CAR-T therapy. Based on previous research [22], we searched the CTA database for those related to LUAD. After a sequence of bioinformatics analyses, we successfully identified an appropriate target antigen, MAGE-A1, from among 876 possible CTAs.

As a member of the MAGE-A antigens, which are the best characterized CTAs, MAGE-A1 is also strictly tumor-specific and is detected in various solid tumors [40–42]. Although MAGE-A1 expression in LC has also been reported [43–45], the detailed and exclusive function of MAGE-A1 in LUAD remains unclear. After MAGE-A1 was screened as the most promising candidate by the aforementioned bioinformatics analyses, a set of investigations was performed to thoroughly examine the characteristics of MAGE-A1 in LUAD. In LUAD cell lines, differential MAGE-A1 expression was detected by qPCR and western blotting tests, and positive staining of MAGE-A1 was witnessed in the cytomembrane by immunofluorescence test. Then IHC analysis in normal human TMA described that MAGE-A1 was dominantly expressed in human testis, not in other human tissues. In LUAD tissue samples, elevated MAGE-A1 expression was also observed and IHC analysis in LUAD TMA further demonstrated that positive MAGE-A1 expression in LUAD was correlated with certain clinical-pathologic characteristics, including tumor diameter and N status. The survival analysis revealed that a high level of MAGE-A1 expression was correlated with unfavorable outcomes of LUAD. All the above data concurred with the studies that showed high expression levels and a prognostic role of MAGE-A1 in LUAD [43, 45, 46].

Although the tumor-promoting activities of MAGE-A1 have been reported in melanoma, possibly due to the activation of the p-C-JUN or ERK-MAPK signaling pathways [47, 48], the biological functions of MAGE-A1 in LUAD have not been fully investigated. Hence, the OEMAGE and shMAGE models in PC9 cells were generated to investigate the malignant behaviors of MAGE-A1 in LUAD. In vitro, the results revealed that OEMAGE significantly increased cell proliferation, migration, and invasion. Conversely, shMAGE critically inhibited cell proliferation, migration, and invasion. In vivo, OEMAGE radically increased the tumor burden, while shMAGE considerably reduced tumor growth. The above data demonstrate that MAGE-A1 expression is functionally important for LUAD development, which is in line with previous studies that described a prominent role played by MAGEs in driving tumorigenesis and progression in LUAD [49–51].

Previously, we produced a human anti-MAGE-A1 scFv and synthesized an immunotoxin [33]. To confirm the legitimacy and suitability of MAGE-A1 as a target antigen for LUAD treatment, we tried to construct a mCAR by adopting the anti-MAGE-A1 scFv and fusing it with CD8α leader, CD8™ and CD137-CD3ζ co-stimulatory domains. The results showed that mCAR was successfully generated and functionally expressed. Then, T cells were collected from a healthy donor, activated by CD3/CD28, expanded by IL-2 and transfected by mCAR lentivirus to produce mCART, which showed high transfection efficiencies and appropriate characteristics. Then, the cytotoxic activity of mCART was evaluated. The LDH results showed that mCART exerted significant cell-lysis activity for MAGE-A1-positive LUAD cells in a dose-dependent manner, accompanied by the release of IFN-γ and IL-2. Our data largely agree with a study reported by Thivyan et al., which illustrated that IFN-γ production could be detected in a positive c-Met expression mesothelioma cell line when it was treated with MET-specific CAR-T [52]. The in vitro results strongly implied that mCART can be activated and expanded in the presence of MAGE-A1-positive LUAD cells and that mCART could specifically destroy LUAD cells by secreting IFN-γ. The cytotoxic effectiveness was improved by increasing the effector to target (E:T) ratio. Moreover, the in vivo experiment thoroughly proved that the tumor-inhibitory competence of mCART for the tumor burdens of mice treated with mCART was much lower than that of mice administered unrelated-CART or T cells and the infiltration ability of mCART into xenograft tumors was also observed.

To date, numerous targets for CAR-T therapy in NSCLC have been evaluated, including EGFR, HER2, MSLN, GPC3, EpCAM, and MUC1 [53]. Nevertheless, MAGEs as targets for CAR-T therapy in LUAD are rare, and prior studies have paid more attention to antitumor vaccines. For instance, MAGE-A3 was once believed to be a potential target in cancer immunotherapy, and a clinical trial demonstrated a promising benefit [54]. The latest research provided negative information regarding MAGE-A3 as the immunotherapeutic adjuvant because it failed to improve the survival of patients with NSCLC [21]. More interestingly, MAGE-A3 was described by an influential study to be essential for cancer cell survival and was shown to play important roles in inducing oncogenic features in noncancerous cells [55]. Therefore, exploration of MAGEs should not be abandoned, and alternative therapeutic strategies should be considered. In the present study, we introduced MAGE-A1 into the CAR-T field and demonstrated the practicability of developing mCART for LUAD treatment.

Intriguingly, a recent study reported a negative attribute of MAGE-A1, showing that it exerted a suppressive, rather than a stimulative role in breast and ovarian cancers. The major reason for this inconsistency is largely due to the disparity of cancer types, which could interfere with the function of c-JUN, FBXW7, and NICD1 and result in the apparently contradictory properties of MAGE-A1 in cancers [56, 57]. Despite this discrepancy, the dominant role of MAGE-A1 in the carcinogenesis of LUAD is well acknowledged, indicating that the scheme for the use of mCART in LUAD treatment is reasonable and convincing.

There are several issues we need to address. We did not employ NSG mice but rather chose athymic nude mice for the in vivo test. Although athymic nude mice are acceptable [10], the optimized and prevailing preclinical model for evaluating CAR-T cells is NSG mice [58]. Moreover, the side effects of mCART in mice were not thoroughly evaluated, such as the injury of important viscera, the potential toxicity to testis, and the release of serum cytokines. In addition, we kept the mice for only 1 month and therefore failed to provide survival data for the mice and data regarding the persistence of mCART. In comparison, Ruella et al. raised the NSG mice for over 8 months so the prognosis of mice and even the long-term immunological memory effect induced by CAR-T cells could be explored [59]. Above all, the mechanism of mCART in LUAD was not elucidated by the present study. For example, immunosuppressive factors in the tumor microenvironment (TME) seem to be a substantial challenge for CAR-T therapy in solid tumors. We need to further inspect how mCART affects the LUAD TME, including checkpoint pathways, cytokines, and other byproducts. In fact, research is ongoing to ameliorate therapeutic effectiveness and to investigate the mechanism of action of mCART in LUAD by our research group. The strategies include the design of dual targeting mCART to enhance tumor antigen recognition, the utilization of cytokine co-expression to improve the survival and infiltrating capacities of mCART, the development of combination therapy with checkpoint inhibitors to boost mCART performance by counteracting immunoevasion, and the construction of hu-CD34-NSG™ and PDX mice models to mimic human TME for mCART mechanism research [60–65].

## Conclusions

Our present study demonstrated that MAGE-A1 is a prospective target in LUAD and that the innovative mCART exerts notable antitumor activity against MAGE-A1-positive LUAD*.* This current study offers a new strategy for LUAD immunotherapy.

## Supplementary information

**Additional file 1:** **Figure S1.** NAA11 was employed to demonstrate the representative expression pattern of 49 CTAs in human tissues, which are marked in red boxes (GTEx Portal database).

**Additional file 2:** **Figure S2.** Demonstration of expression of compartment and confidence for four CTAs (MAGE-A1, ADAM2, TEX101 and Clorf49) (GeneCard database).

**Additional file 3:** **Figure S3.** Comparison of tumor weight of xenograft tumors in WT, shMAGE, shCT, OEMAGE, OECT tumors at 48 days after cell inoculation. * Significant difference in tumor weight in the OEMAGE and shMAGE groups compared with that in the WT group.

**Additional file 4:** **Figure S4.** Titer detection of lentivirus transfection and determination of optimum titer in 10^− 2^, 10^− 3^, 10^− 4^, and 10^− 5^ different concentrations of lentivirus .The lentivirus titer was 1 × 10^8^ TU/mL.

**Additional file 5:** **Figure S5.** A. The growth curve of xenograft tumors when treated with mCART, unrelated-CART and T. The administration of mCART illustrated the most significant tumor-inhibitory effectiveness. * Significant difference in tumor volume in the mCART group compared with the T group. B. Body weight of xenograft nude mice in three treated groups (mCART, unrelated-CART and T) showed no significant difference.

**Additional file 6.** Detailed data of CTA screen.

**Additional file 7:** **Table S2.** Primer and siRNA sequences.

**Additional file 8:** **Table S3.** MAGE-A1-scFv amino acid sequence.

## Data Availability

All data generated or analyzed during this study are included in the manuscript and its supplementary information files.

## Abbreviations

CAR-T: Chimeric antigen receptor-engineered T
CTAs: Cancer/testis antigens
EGFR: Epidermal growth factor receptor
FACS: Fluorescence-activated cell sorting
LC: Lung cancer
LUAD: Lung adenocarcinoma
mCART: MAGE-A1-CAR-T cell
NSCLC: Non-small cell lung cancer
OEMAGE: MAGE-A1 overexpression
OS: Overall survival
PBMC: Peripheral blood mononuclear cell
scFv: Single-chain variable fragment
shMAGE: MAGE-A1 knockdown
shRNA: Short-hairpin RNA
SPF: Specific pathogen-free
TAAs: Tumor-associated antigens
TCGA: The Cancer Genome Atlas
TMA: Tissue microarrays
TME: Tumor microenvironment
