# Supplementary figures and images for: MAGE-A1 in lung adenocarcinoma as a promising target of chimeric antigen receptor T cells
Source: J Hematol Oncol. 2019 Oct 22;12:106. doi: 10.1186/s13045-019-0793-7 (PMC6805483; doi:10.1186/s13045-019-0793-7)

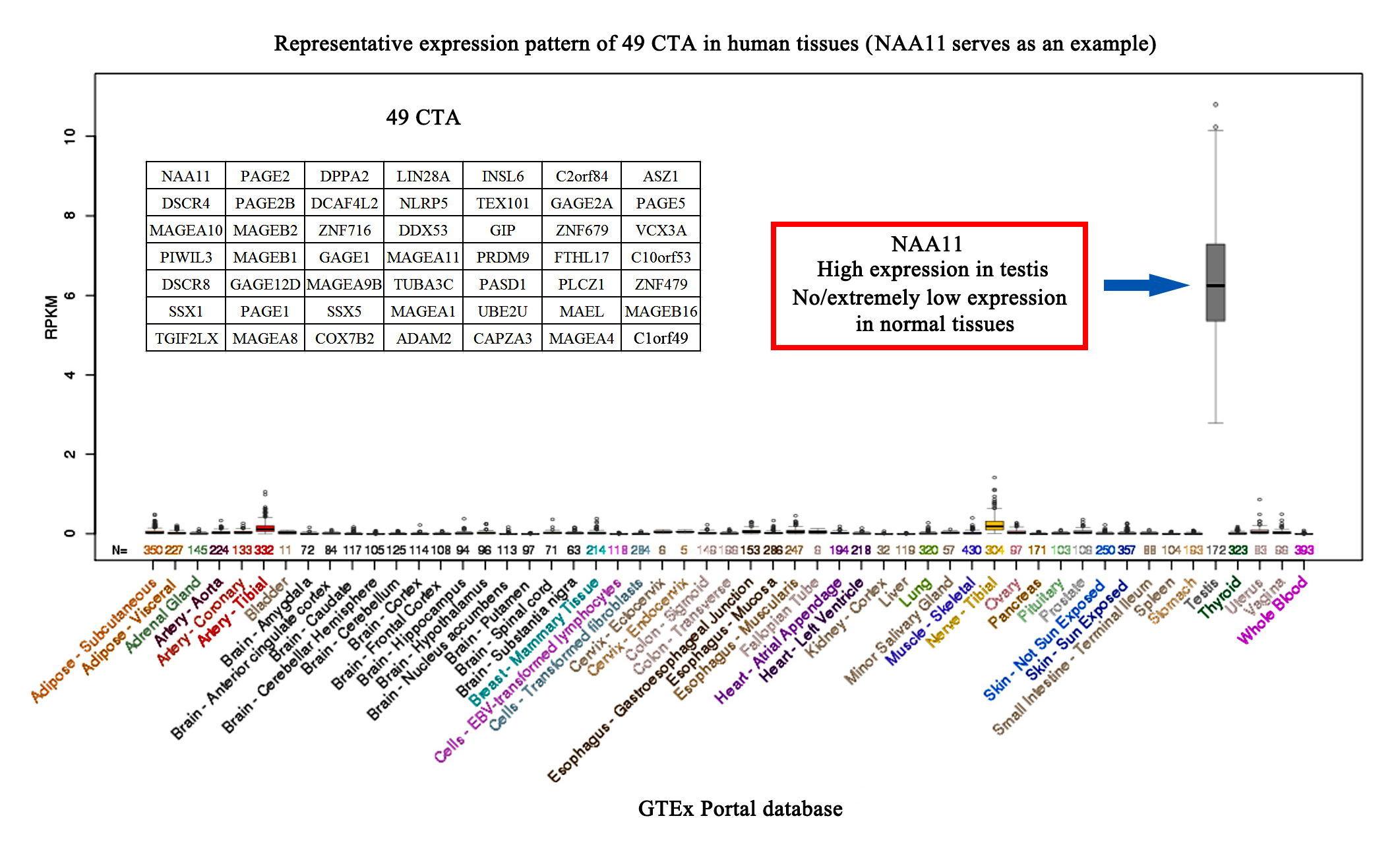

Supplement: Supplementary file 1 — Additional file 1: Figure S1. NAA11 was employed to demonstrate the representative expression pattern of 49 CTAs in human tissues, which are marked in red boxes (GTEx Portal database). [file 13045_2019_793_MOESM1_ESM.jpg]

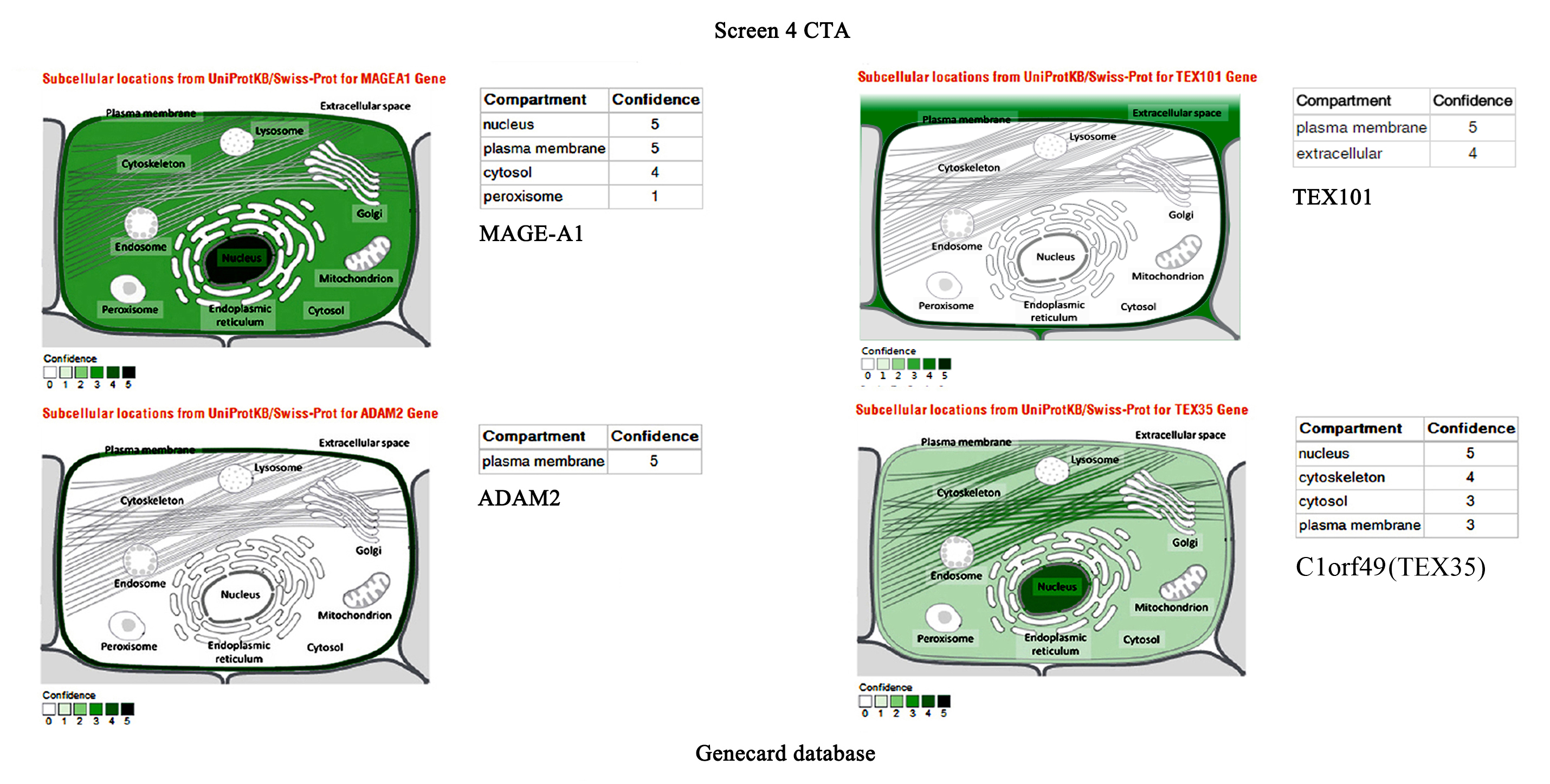

Supplement: Supplementary file 2 — Additional file 2: Figure S2. Demonstration of expression of compartment and confidence for four CTAs (MAGE-A1, ADAM2, TEX101 and Clorf49) (GeneCard database). [file 13045_2019_793_MOESM2_ESM.jpg]

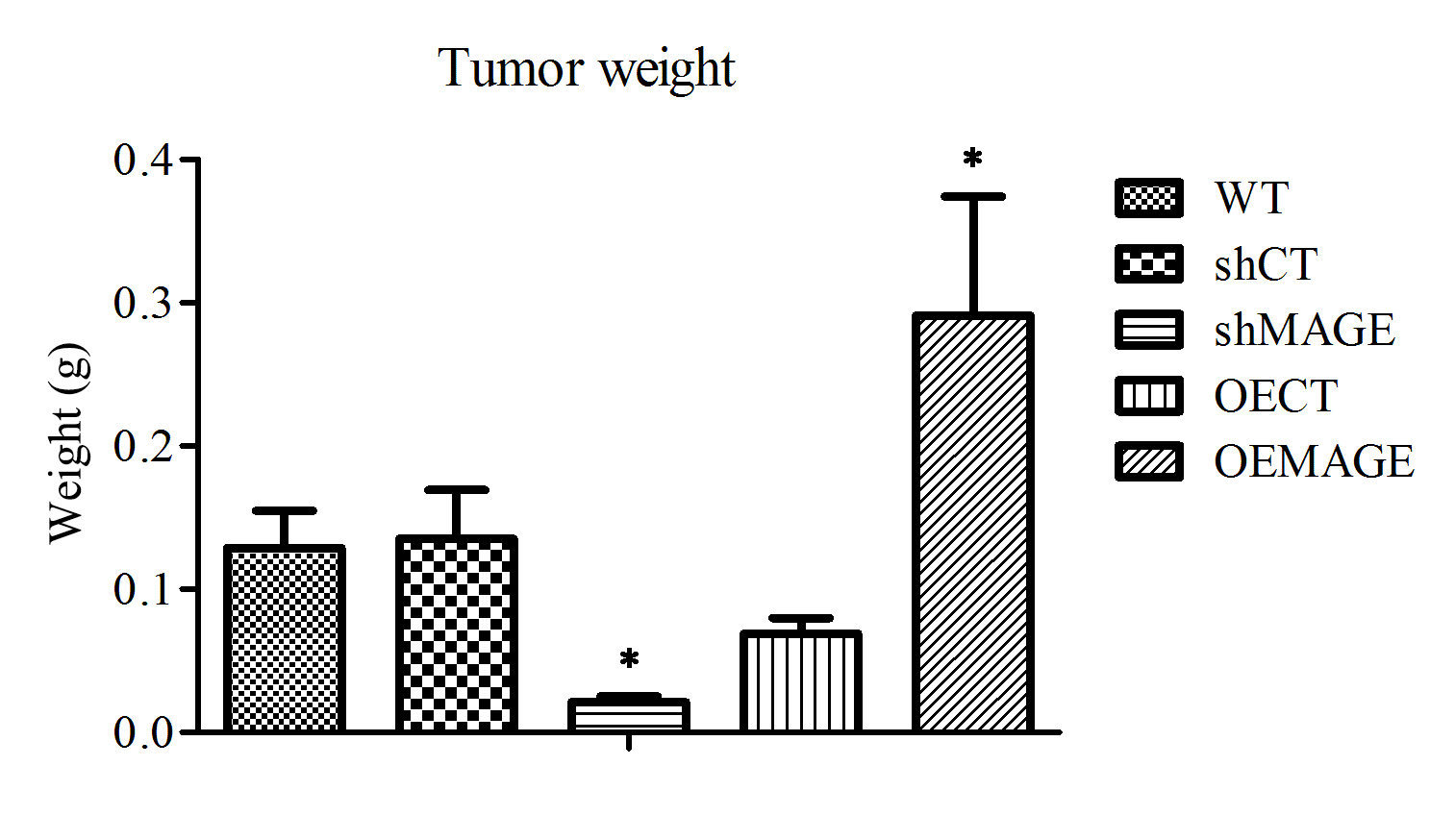

Supplement: Supplementary file 3 — Additional file 3: Figure S3. Comparison of tumor weight of xenograft tumors in WT, shMAGE, shCT, OEMAGE, OECT tumors at 48 days after cell inoculation. * Significant difference in tumor weight in the OEMAGE and shMAGE groups compared with that in the WT group. [file 13045_2019_793_MOESM3_ESM.jpg]

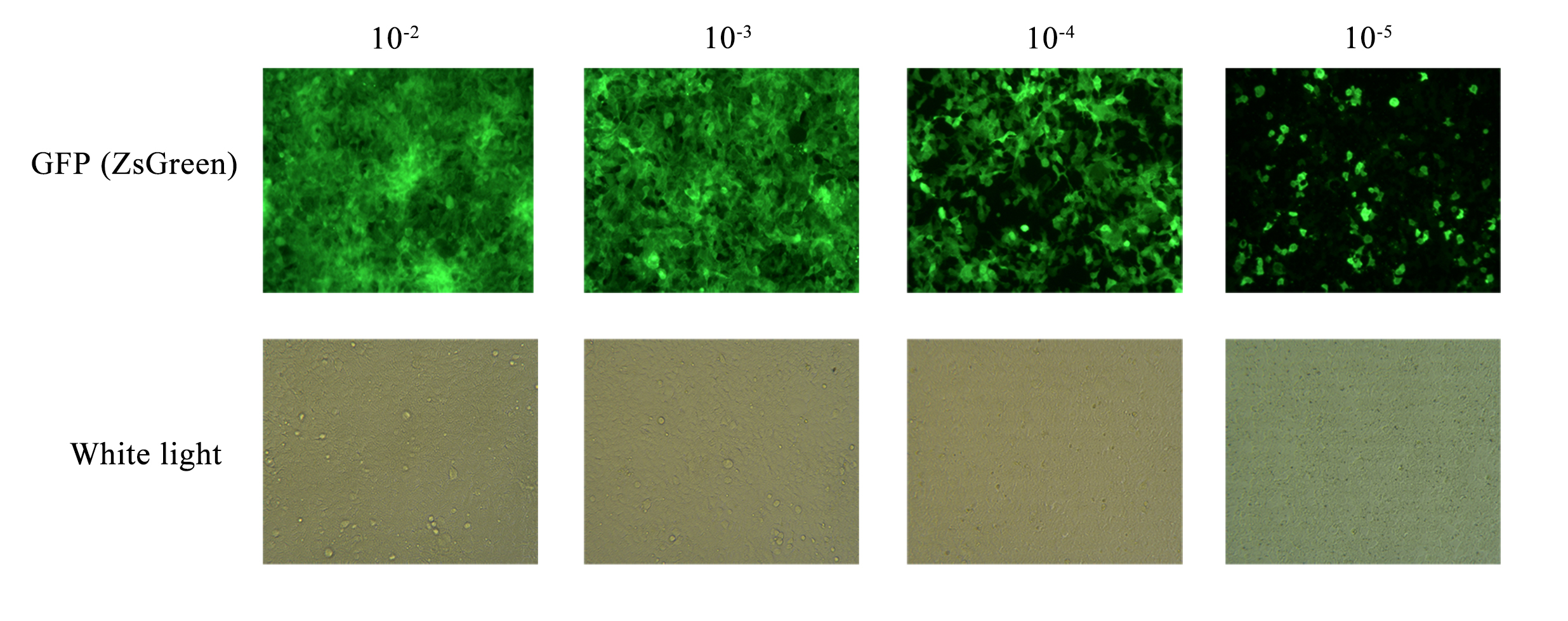

Supplement: Supplementary file 4 — Additional file 4: Figure S4. Titer detection of lentivirus transfection and determination of optimum titer in 10− 2, 10− 3, 10− 4, and 10− 5 different concentrations of lentivirus .The lentivirus titer was 1 × 10^8^ TU/mL. [file 13045_2019_793_MOESM4_ESM.jpg]

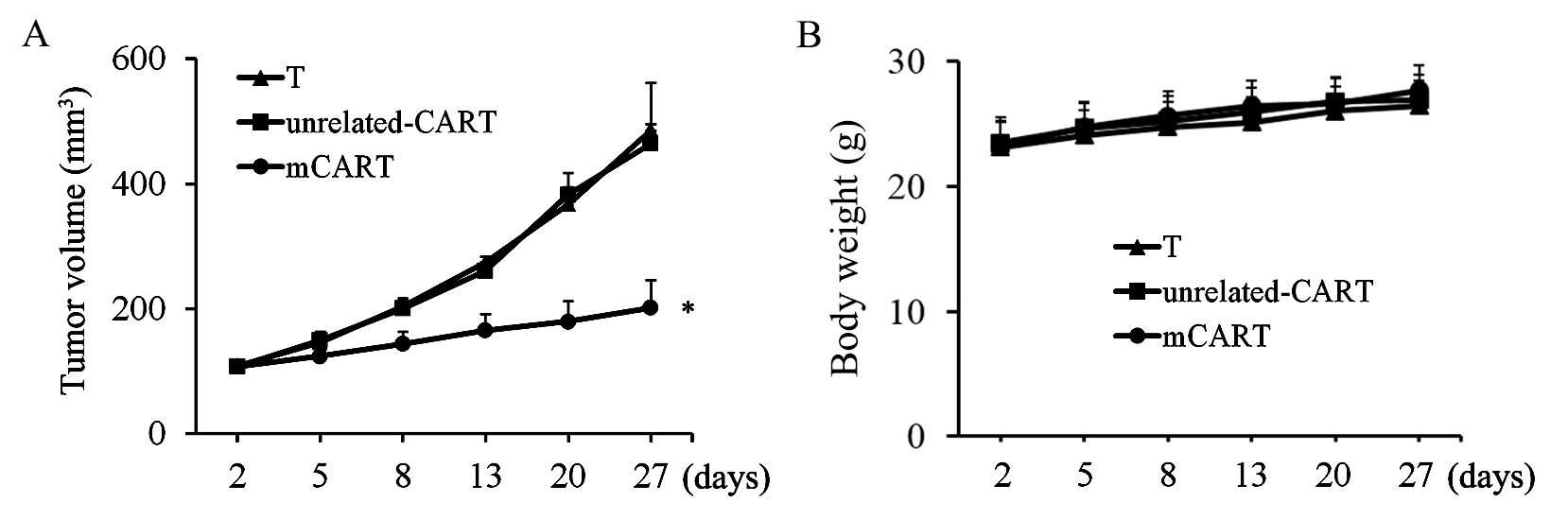

Supplement: Supplementary file 5 — Additional file 5: Figure S5. A. The growth curve of xenograft tumors when treated with mCART, unrelated-CART and T. The administration of mCART illustrated the most significant tumor-inhibitory effectiveness. * Significant difference in tumor volume in the mCART group compared with the T group. B. Body weight of xenograft nude mice in three treated groups (mCART, unrelated-CART and T) showed no significant difference. [file 13045_2019_793_MOESM5_ESM.jpg]
