# Supplementary material for: MAGE-A1 in lung adenocarcinoma as a promising target of chimeric antigen receptor T cells
Source: J Hematol Oncol. 2019 Oct 22;12:106. doi: 10.1186/s13045-019-0793-7 (PMC6805483; doi:10.1186/s13045-019-0793-7)
Supplement: Supplementary file 7 — Additional file 7: Table S2. Primer and siRNA sequences. [file 13045_2019_793_MOESM7_ESM.docx]

Table S2. Primer and siRNA sequences

| Primer types | Primer sequences |
| --- | --- |
| MAGE-A1 forward | 5’- CAG CAT TTC TGC CTT TGT -3’ |
| MAGE-A1 reverse | 5’- GCC TTT CCC ACT ACC ATC -3’ |
| MAGE-A1 siRNA1 | 5’- GCA AAG CCT CTG AGT CCT T -3’ |
| MAGE-A1 siRNA2 | 5’- GCT CCT GAG GAG GAA ATC T -3’ |
| MAGE-A1 siRNA3 | 5’- CCT CGC TGA AAC CAG CTA T -3’ |
| MAGE-A1 siRNA-NC | 5’- TTC TCC GAA CGT GTC ACG T -3’ |
| β-actin forward | 5’- CTC CAT CCT GGC CTC GCT GT -3’ |
| β-actin reverse | 5’- GCT GCT ACC TTC ACC GTT CC -3’ |
| GAPDH forward | 5’ -TGC ACC ACC AAC TGC TTA GC -3’ |
| GAPDH reverse | 5’- GGC ATG GAC TGT GGT CAT GA -3’ |
