# Supplementary material for: MAGE-A1 in lung adenocarcinoma as a promising target of chimeric antigen receptor T cells
Source: J Hematol Oncol. 2019 Oct 22;12:106. doi: 10.1186/s13045-019-0793-7 (PMC6805483; doi:10.1186/s13045-019-0793-7)
Supplement: Supplementary file 8 — Additional file 8: Table S3. MAGE-A1-scFv amino acid sequence. [file 13045_2019_793_MOESM8_ESM.docx]

Table S3. MAGE-A1-scFv amino acid sequence

| MAGE-A1-scFv amino acid sequence |
| --- |
| >MAGE-A1 VH  EVQLLESGGGLVQPGGSLRLSCAASGFTFSSYAMSWVRQAPGKGLEWVSAISGSGGSTYYADPVKGRFTISRDNSKNTLYLQMNSLRAEDTAVYYCAKLIHDFDYWGQGTLVTVSS |
| >MAGE-A1 VK  DIQMTQSPSSLSASVGDRVTITCRASQSISSYLNWYQQKPGKAPKLLIYRASALQSGVPSRFSGSGSGTDFTLTISSLQPEDFATYYCQQSRKTPHTFGQGTKVEIK |
